# Changes in Ileal Microbial Composition and Microbial Metabolism by an Early-Life Galacto-Oligosaccharides Intervention in a Neonatal Porcine Model

**DOI:** 10.3390/nu11081753

**Published:** 2019-07-30

**Authors:** Shiyi Tian, Jue Wang, Hu Yu, Jing Wang, Weiyun Zhu

**Affiliations:** National center for International Research on Animal Gut Nutrition, Jiangsu Key Laboratory of Gastrointestinal Nutrition and Animal Health, Laboratory of Gastrointestinal Microbiology, National Experimental Teaching Demonstration Center of Animal Science, College of Animal Science and Technology, Nanjing Agricultural University, Nanjing 210095, China

**Keywords:** galacto-oligosaccharides, suckling piglets, microbial composition, microbial metabolites, endocrine peptides, inflammatory cytokines, antimicrobial peptides

## Abstract

Galacto-oligosaccharides (GOS), functional oligosaccharides with natural characteristics, are important active substances in milk that play an important role in the development of intestinal microbiota and the immune system of newborns. The intestinal maturation of piglets resembles that of human newborns and infants. Therefore, we used the newborn piglet model to study the effects of early-life GOS intervention. Six litters of neonatal piglets (10 piglets per litter) with the same average birth weight were divided into control (CON) and GOS (GOS) groups in each litter. Piglets in the GOS group were given 10 mL of GOS solution daily during the first week after birth, while piglets in the CON group were given the same dose of physiological saline orally. One pig per group from each litter was euthanized on day 8 and day 21. Results revealed that ileal microbiota composition was significantly enriched in *Lactobacillus* and unclassified Lactobacillaceae, and reduced in *Clostridium sensu stricto* on day 8 and day 21 after GOS intervention. Additionally, *Escherichia* significantly decreased on day 21 following the early-life GOS intervention. Moreover, the content of microbial metabolites, endocrine peptides, and the mRNA expression of anti-inflammatory cytokines and antimicrobial peptides increased in the GOS group. These findings provide guidelines for early prebiotic supplementation for lactating newborns.

## 1. Introduction

The intestine has the highest number of immune cells and the highest diversity of microbiota that play a crucial role in nutrient supply and maintaining host health [1]. Mammalian intestinal microbiota are indispensable in preventing infectious diseases, maintaining intestinal morphology, digesting and absorbing nutrients, and regulating immunity [2,3,4]. The structure and function of the mucosal immune system in the intestine develop right after birth, accompanied by a fast colonization of intestinal microbiota [5]. There is evidence that the early colonization of the intestinal microbiota affects the immune maturation process [6,7]. In addition, studies have shown that host species-specific microbiota are necessary for immune system development [8]. Therefore, the early colonization of the intestine by microbiota determines the immune capacity of the host in the later stages of life [9]. Since intestinal microbiota are dynamic and impressionable to environmental conditions in early life [5], modulating the intestinal microbiota development through dietary strategies has become an attractive approach to maintain host health. 

One modulating strategy is to use dietary prebiotics, indigestible food ingredients that resist absorption in the intestine and are selectively fermented by intestinal microbiota. The prebiotics can stimulate the activity of beneficial intestinal bacteria, including *Lactobacillus* and *Bifidobacteria* [10]. After dietary prebiotics enter the gut, beneficial intestinal bacteria hydrolyze them, and produce nutrients and energy, which in turn promote the activity of beneficial intestinal bacteria [11]. At the same time, intestinal bacteria ferment dietary prebiotics to produce short-chain fatty acids (SCFAs) and lactate. The SCFAs are absorbed by enterocytes and have beneficial effects on host health [12]. In addition to SCFAs, lactate is also a major fermentation product of carbohydrate metabolism. It reduces pH in the intestine and inhibits the activity of pathogenic bacteria [13]. Galacto-oligosaccharides (GOS) are natural oligosaccharides and important active substances in milk [14]. As prebiotics, GOS directly improve the intestinal barrier, reduce the colonization of pathogenic bacteria in the intestine, and promote intestinal health [15]. They also regulate the composition of intestinal microbiota, increase the colonization of beneficial bacteria such as *Bifidobacterium* and *Lactobacillus*, and reduce the colonization of harmful bacteria like *Escherichia coli* [16]. In addition, GOS can improve lipid metabolism [17], increase the absorption of mineral elements, and prevent bone loss [18].

It has been reported that humans and pigs share a high degree of physiological similarity in digestive and associated metabolic processes [19]. Pigs’ intestinal microbial ecosystems are similar to humans’ because pigs are human-sized omnivores with similar nutritional requirements [19]. In addition, pigs are often used as experimental models for assessing interactions between microbiota and health, as they exhibit human-like symptoms such as necrotizing enterocolitis (NEC) and partial weaning diarrhea [19]. Taken together, the intestinal development and nutritional requirements of piglets after birth are more similar to those of human infants in many aspects [19]. Thus, it is important to evaluate the impact of GOS on microbial colonization and metabolism of suckling piglets. Since the ileum is a site where interactions between mucosal cells, microbiota, and nutrients occur [20], the current study investigated the effects of early-life GOS intervention (during the seven days after birth) on ileal microbiota, microbial metabolism, and intestinal health of suckling piglets.

## 2. Materials and Methods 

### 2.1. Animal Trial

Animal ethics approval for this study was obtained from the Animal Experiment Committee of Nanjing Agricultural University, in accordance with the Regulations for the Administrations of Affairs Concerning the Experimental Animals. All methods were performed in accordance with the approved guidelines and regulations.

Six litters of neonatal piglets (Duroc × Landrace × Large White) were selected, 10 piglets per litter. Then, the piglets per litter were evenly and randomly assigned to control groups (CON) and GOS groups (GOS) with similar body weight. GOS of 90% purity were purchased from Quantum Hi-Tech Biological co., Ltd. (Jiangmen, China). The composition of GOS is displayed in Figure S1 and the information of carbohydrate components in GOS sample is displayed in Table S1. During the first week after birth, the GOS group was orally administrated with 10 mL GOS solution (1 g/kg bodyweight) per day [21,22,23], and the CON group was treated with the same dose of physiological saline. Piglets had free access to sow milk and water at all times throughout the experimental period. Health status was monitored daily, and all piglets were kept healthy during the experimental period.

On day 8 and day 21, six piglets from each group were euthanized. After the piglets were sacrificed, ileal digesta were collected for determination of intestinal microbiota and microbial metabolites. The pH of ileal digesta was measured using a pH meter. Ileal mucosa was collected for further analysis.

### 2.2. Microbiota Profiling

Total bacterial DNA was extracted from the ileal digesta following a previous study [24]. A universal primer was used for 16S rRNA gene amplification [25]. The quality of the amplicons was detected using gel electrophoresis, and the amplicons were purified using AxyPrep DNA Gel Extraction Kit (Axygen Biosciences, Union City, CA, USA). Purified amplicons were pooled in equimolar concentrations for subsequent sequencing. The raw reads were uploaded to the NCBI Sequence Read Archive database (Accession number SRP165134).

As described in previous study, Raw fastQ files were de-multiplexed and quality-filtered using QIIME (version 1.70) [25]. Operational taxonomic units (OTUs) were clustered with a 97% similarity cut-off using UPARSE (version 7.1), and chimeric sequences were identified and removed using UCHIME [26]. The most abundant sequences within each OTU were designated as representative sequences, which were classified using the Ribosomal Database Project (RDP) classifier [27]. The diversity indices and principal coordinate analysis (PCoA) were assessed using MOTHUR v.1.29.0 as described in previous studies [28,29]. 

### 2.3. Microbial Metabolites

The concentrations of short-chain fatty acids (SCFAs) in the ileal digesta were determined using gas chromatography (GC) method as described in previous study [25]. The lactate concentration was measured using a commercial kit (Nanjing Jiancheng Bioengineering Institute, Nanjing, China).

### 2.4. RT-PCR

Total RNA was isolated using TriZOL (Invitrogen, Carlsbad, CA, USA) according to the manufacturer’s protocol. After the purity and integrity of RNA were detected, the total RNA was reverse-transcribed to cDNA using a PrimeScript RT reagent kit (Takara Biotechnology (Dalian) Co., Ltd., Dalian, China) according to the manufacturer’s protocol. The genes primers—interleukin-8 (*IL-8*), interleukin-10 (*IL-10*), porcine β-defensin-1 (*PBD-1*), porcine β-defensin-3 (*PBD-3*), regenerating islet derived protein-3γ (*Reg-3γ*), and housekeeping genes (*β-actin*)—are listed in Additional File 1: Table S2. According to the manufacturer’s protocol, qRT-PCR reactions were performed on an Applied Biosystems 7300 Real-Time PCR system using a SYBR Premix Ex Taq^TM^ (Tli RnaseH Plus) qPCR kit (Takara Biotechnology (Dalian) Co., Ltd.). Expression levels were calculated using the 2^−ΔΔCt^ method [30] and normalized to the housekeeping gene *β-actin*. 

### 2.5. Endocrine Peptides

The determination of glucagon-like peptide-1 (GLP-1), glucagon-like peptide-2 (GLP-2), insulin-like growth factor 1 (IGF-1), and epidermal growth factor (EGF) levels in intestinal mucosa was conducted using the ProcartaPlex™ multiplex immunoassay kit (Luminex, Austin, TX, USA) as previously reported [31]. 

### 2.6. Statistical Analysis

Results were expressed as means ± SEM. The Shapiro–Wilk test was used to evaluate the normality of the distribution of the data. Data with normal distribution were analyzed by the student’s *t*-test procedure, and the Mann–Whitney *U*-test was used to analyze data with non-normal distribution. Statistical significance was defined as *P* < 0.05, whereas *P* values between 0.05 and 0.10 were considered as indicative of a trend. The *R* package of “Hmisc” was used for calculating the Spearman’s correlation coefficient. 

## 3. Results

### 3.1. Effects of the Early-Life GOS Intervention on the Diversity of the Ileal Digesta Microbiota

**Figure 1:**
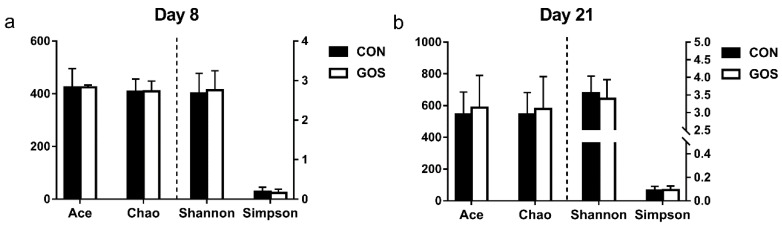
Effects of early-life galacto-oligosaccharides (GOS) intervention on the diversity of ileal microbiota at the 3% dissimilarity level of suckling piglets. (**a**) The diversity of ileal microbiota on day 8; (**b**) the diversity of ileal microbiota on day 21. Values are expressed as means ± SEM, CON, a control group; GOS, a galacto-oligosaccharides intervention group.

**Figure 2:**
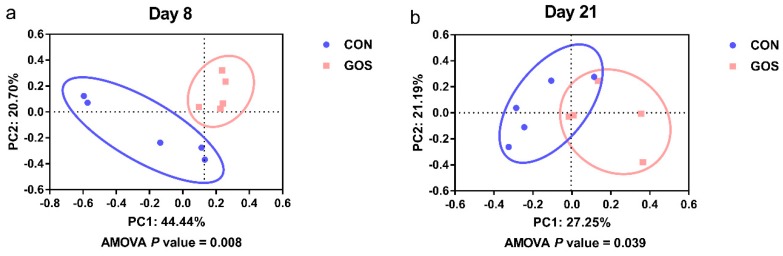
Principle coordinate analysis of ileum samples in the CON and GOS group. (**a**) The principle coordinate analysis of ileal microbiota on day 8; (**b**) the principle coordinate analysis of ileal microbiota on day 21. The percentage of variation explained by PC1 and PC2 are indicated in the axis. CON, a control group; GOS, a galacto-oligosaccharides intervention group.

Sequence data showed that GOS affected the microbial composition of ileal digesta. Samples with <25,000 sequence valid reads were excluded from the analysis, resulting in *n* = 5 for every group on day 8 and day 21. In addition, the number of average raw sequences detected in each group was more than 27,782 valid sequences (Table S3). Results of gamma diversity shows that OTU clustering (97% cutoff) yielded a total of 1492 OTUs for the entire dataset. As shown in Figure 1, the diversity indices (Shannon and Simpson) and richness estimators (Ace and Chao) of ileal digesta microbiota were similar in the CON and GOS groups on day 8 and day 21. As shown in Figure 2, the PCoA analysis revealed that the ileal microbiota composition was significantly altered after early intervention with GOS on day 8 and day 21, with an evident separation between the CON and GOS groups (AMOVA, *P* < 0.05).

### 3.2. Effects of the Early-Life GOS Intervention on the Abundance of Ileal Microbiota

**Figure 3:**
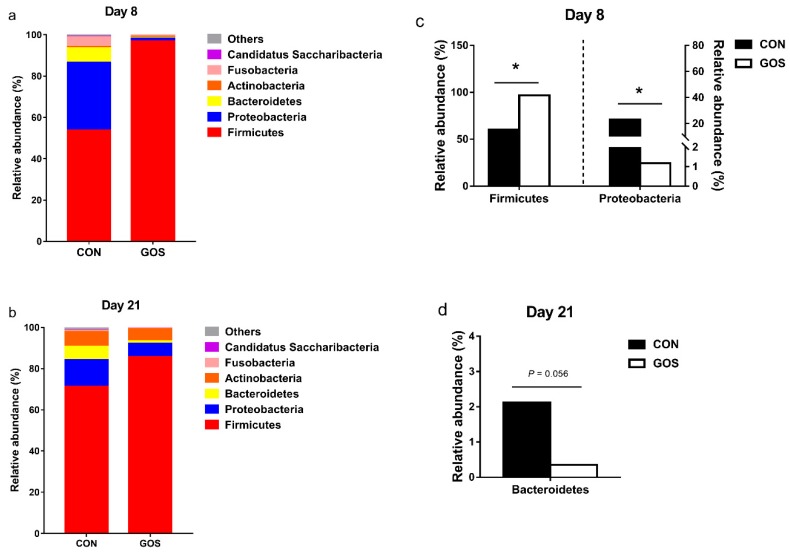
Effects of early-life galacto-oligosaccharides (GOS) intervention on the phylum-level composition. (**a**,**b**) The phylum-level composition of average relative abundance ileal microbiota in suckling piglets; (**c**,**d**) the changes in the abundance of bacterial phyla found in ileum. The values are expressed as the medians, with five piglets per group. The “*” indicates a significant difference (*P* < 0.05) between the CON and GOS group (Mann–Whitney *U*-test). CON, a control group; GOS, a galacto-oligosaccharides intervention group.

The bacterial composition was assessed at different taxonomic levels. At the phylum level, the dominant bacterial groups were Firmicutes (day 8, CON group: 53.65%, GOS group: 96.92%; day 21, CON group: 71.21%, GOS group: 85.73%), Proteobacteria (day 8, CON group: 32.98%, GOS group: 1.24%; day 21, CON group: 12.99%, GOS group: 6.39%), and Bacteroidetes (day 8, CON group: 6.84%, GOS group: 0.49%; day 21, CON group: 6.59%, GOS group: 1.21%); these were followed by the bacteria from phyla Actinobacteria, Fusobacteria, and Candidatus Saccharibacteria (Figure 3a,b, Table S4). The abundance of Firmicutes increased, and the abundance of Proteobacteria decreased (*P* < 0.05) in the GOS group compared with those in the CON group on day 8 (Figure 3c). In contrast, the abundance of Bacteroidetes tended to decrease (*P* = 0.056) in the GOS group compared with the CON group on day 21 (Figure 3d).

**Figure 4:**
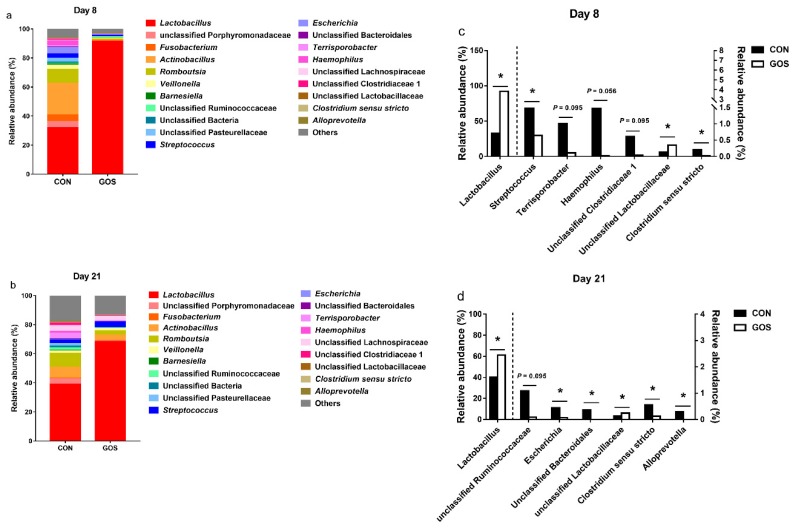
Effects of early-life galacto-oligosaccharides (GOS) intervention on the genus-level composition. (**a**,**b**) The genus-level composition of average relative abundance ileal microbiota in suckling piglets, (**c**,**d**) the changes in the abundance of bacterial genera found in ileum. The values are expressed as the medians, with five piglets per group. The “*” indicates a significant difference (*P* < 0.05) between the CON and GOS group (Mann–Whitney *U*-test). CON, a control group; GOS, a galacto-oligosaccharides intervention group.

At the genus level, the most dominant genus was *Lactobacillus* (day 8, CON group: 31.95%, GOS group: 91.49%, day 21, CON group: 39.12%, GOS group: 68.43%). In addition, *Lactobacillus* (31.95%), *Actinobacillus* (21.67%), *Romboutsia* (9.65%), *Fusobacterium* (4.55%), unclassified Porphyromonadaceae (4.36%), *Escherichia* (4.34%), *Haemophilu* (3.44%), *Streptococcus* (3.37%), *Veillonella* (2.72%), and unclassified Pasteurellaceae (2.67%) were the most abundant genera (>1%) in the CON group, while *Lactobacillus* (91.49%) was the most abundant genera (>1%) in the GOS group on day 8. On day 21, *Lactobacillus* (39.12%), *Romboutsia* (9.58%), *Actinobacillus* (7.62%), unclassified Lachnospiracea (3.95%), *Terrisporobacter* (3.87%), unclassified Porphyromonadaceae (3.59%), *Streptococcus* (2.45%), unclassified Pasteurellaceae (1.91%), unclassified Clostridiaceae 1 (1.62%), unclassified Ruminococcaceae (1.56%), *Veillonella* (1.40%), *Haemophilus* (1.19%), and unclassified Bacteria (1.07%) were the most abundant genera (>1%) in the CON group, while *Lactobacillus* (68.43%), *Actinobacillus* (3.96%), *Streptococcus* (3.95%), *Romboutsia* (2.91%), unclassified Lachnospiraceae (2.89%), and *Veillonella* (1.44%) were the most abundant genera (>1%) in the GOS group (Figure 4a,b, Table S5). The abundance of *Lactobacillus* and unclassified Lactobacillaceae increased in the GOS group (*P* < 0.05) compared with the CON group on day 8 (Figure 4c). In addition, the abundance of *Streptococcus* and *Clostridium sensu stricto* decreased in the GOS group compared to the CON group (*P* < 0.05) (Figure 4c). The abundance of *Terrisporobacter* (*P* = 0.095), *Haemophilus* (*P* = 0.056), and unclassified Clostridiaceae 1 (*P* = 0.095) displayed a decreasing trend in the GOS group compared with the CON group (Figure 4c). On the other hand, the abundance of *Lactobacillus* and unclassified Lactobacillaceae was higher in the GOS group than that in the CON group (*P* < 0.05) on day 21 (Figure 4d). The abundance of *Escherichia*, unclassified Bacteroidales, *Clostridium sensu stricto,* and *Alloprevotella* was also lower in the GOS group than in the CON group (*P* < 0.05) (Figure 4d). Furthermore, the abundance of unclassified Ruminococcaceae tended to decrease in the GOS group compared with the CON group (*P* = 0.095) (Figure 4d).

### 3.3. Effects of the Early-Life GOS Intervention on pH Value, SCFAs, and Lactate Concentrations in Ileal Digesta

To explore the metabolic alterations associated with an early-life GOS intervention, the metabolites in the ileal digesta of the GOS-fed piglets and control piglets were determined. The pH value and SCFA and lactate concentrations in the ileal digesta are listed in Table 1. The pH value of the ileal digesta decreased in the GOS group compared with the CON group on day 8 (*P* < 0.05). However, there was no statistical difference in the pH of the ileal digesta in the CON and GOS groups on day 21 (*P* > 0.05). Regarding SCFAs, the piglets in the GOS group had significantly higher concentrations of propionate, butyrate, and valerate in their ileal digesta compared to those in the CON group (*P* < 0.05) on day 8. The concentration of acetate in the ileal digesta of the piglets in the GOS group tended to increase compared with that in the CON group (*P* = 0.099). In addition, GOS piglets had a greater concentration of butyrate compared with CON piglets (*P* < 0.05) on day 21. The GOS group had a higher concentration of lactate on day 8 compared with the CON group (*P* < 0.05). However, no difference was observed in the detected lactate concentration and pH value between the CON and GOS groups on day 21.

### 3.4. Effects of the Early-Life GOS Intervention on the mRNA Expression of Cytokines and Antimicrobial Peptide in the Ileal Mucosa

**Figure 5:**
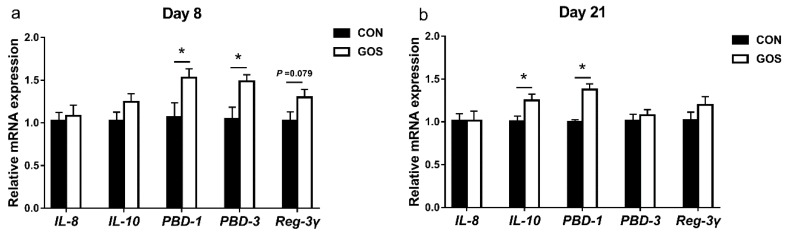
The relative mRNA expression of the ileal inflammatory cytokines and antimicrobial peptides in suckling piglets on day 8 (**a**) and day 21 (**b**). Values are expressed as means ± SEM, *n* = 6. The “*” indicates a significant difference (*P* < 0.05) between the CON and GOS group. CON, a control group; GOS, a galacto-oligosaccharides intervention group.

We also analyzed the mRNA expression of the cytokines and antimicrobial peptide in the ileal mucosa (Figure 5). On day 8, the mRNA expression of *PBD-1* and *PBD-3* was higher in the GOS group than that in the CON group (*P* < 0.05), and the mRNA expression of *Reg-3γ* displayed an increasing trend to increase in GOS group (*P* = 0.079) without any difference in the mRNA expression of *IL-8* and *IL-10* (*P* > 0.05). On day 21, the piglets in GOS group had a higher expression level of *IL-10* and *PBD-1* (*P* < 0.05) in the ileal mucosa than the piglets in the CON group. In addition, there was no difference in the mRNA expression of *IL-8*, *PBD-3,* and *Reg-3γ* in ileal mucosa of piglets between the two groups (*P* > 0.05).

### 3.5. Effects of the Early-Life GOS Intervention on the Concentration of Endocrine Peptides in the Ileal Mucosa

The effects of the early-life GOS intervention on the concentration of endocrine peptides is illustrated in Table 2. On day 8, compared with the CON group, the GOS increased the concentration of GLP-1 (*P* < 0.05), and had a tendency to increase the concentration of EGF (*P* = 0.059). On day 21, no significant difference was observed between the two groups (*P* > 0.05).

### 3.6. Correlation Analysis between the Ileal Microbiota and the Metabolites, the Ileal Cytokines’ Expression, Antimicrobial Peptides’ Expression, and Ileal Endocrine Peptides Levels

**Figure 6:**
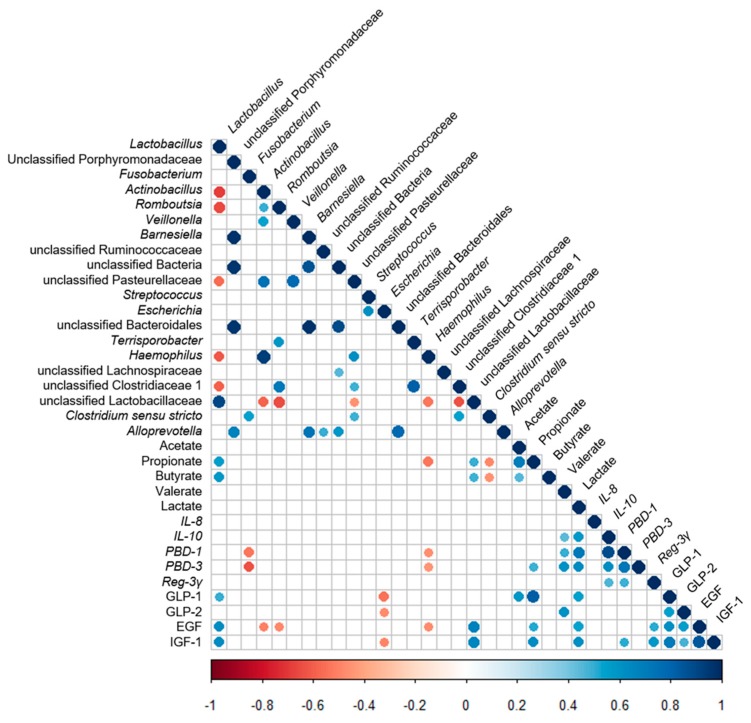
Correlation analysis between the ileal microbiota and the metabolites, the ileal cytokines’ expression, antimicrobial peptides’ expression, and ileal endocrine peptides levels. The R package of “corroplot” was used for generating the heat maps. The blue represents a significant positive correlation, and the red represents a significant negative correlation.

A Spearman’s correlation analysis was used to determine the correlation among the distributions of the ileal cytokine mRNA expression, antimicrobial peptide mRNA expression, ileal endocrine peptides levels, the ileal microbiota, and metabolites. The resulting metabolic association heatmap (Figure 6) indicated positive or negative correlations between the microbiota and the metabolites, cytokines, antimicrobial peptides, and intestinal growth factors. First, when the correlation between the microbiota was considered, the abundance of *Lactobacillus* displayed a strong positive correlation with the abundance of unclassified *Lactobacillaceae* (*P* < 0.05) and a negative correlation with the abundance of *Actinobacillus*, *Romboutsia*, unclassified Pasteurellaceae, *Haemophilus,* and unclassified Clostridiaceae 1 (*P* < 0.05). Second, when the correlations between ileal microbiota and metabolites, ileal cytokine mRNA expression, antimicrobial peptide mRNA expression, and ileal intestinal growth factor levels were considered, the abundance of unclassified Lactobacillaceae was similar to *Lactobacillus*, and it exhibited a positive correlation with the concentration of SCFAs and intestinal growth factors (*P* < 0.05). The abundance of *Fusobacterium* was negatively correlated with the mRNA expression of *PBD-1* and *PBD-3* (*P* < 0.05). The abundance of *Actinobacillus*, *Romboutsia,* and *Escherichia* was negatively correlated with the concentration of intestinal growth factors (*P* < 0.05). The abundance of *Haemophilus* showed a strong negative correlation with the concentrations of propionate and EGF as well as the mRNA expression of *PBD-1* and *PBD-3* (*P* < 0.05). The abundance of *Clostridium sensu stricto* exhibited a negative correlation with the concentrations of propionate and butyrate (*P* < 0.05). Third, when the correlations between metabolites and cytokines, as well as antimicrobial peptides were considered, the concentrations of SCFAs and lactate were positively correlated with the mRNA expression of cytokines and antimicrobial peptides (*P* < 0.05). Furthermore, the SCFAs and lactate concentrations were also positively correlated with the mRNA expression of endocrine peptides (*P* < 0.05).

## 4. Discussion

Dietary nutrients, such as probiotics and prebiotics, play an important role in constructing the composition of intestinal microbiota [13,32]. GOS are prebiotics recognized for their health benefits [15]. GOS can alter the intestinal microbiota, which in turn improve animal performance and health [15]. Our previous study suggested that early-life GOS intervention enhances gut barrier function in a neonatal piglet model [29,31]. In the present study, we investigated the change of the ileal microbiota composition, microbial metabolism, and ileal function after an early-life GOS intervention. The early-life GOS intervention markedly affected the ileal microbiota composition by increasing the abundance of *Lactobacillus*, reducing the abundance of *Escherichia,* and significantly increasing the concentration of SCFAs and lactate. In addition, the mRNA expression of antimicrobial peptides such as *PBD-1* and *PBD-3* increased after an early-life intervention. Moreover, the endocrine peptides also increased after an early-life GOS intervention. These results indicated a significant impact of the early-life GOS intervention on ileal microbiota and microbial metabolites of suckling piglets.

High-throughput sequencing analysis revealed significant differences in ileal microbiota between the CON and GOS groups. The comparison of alpha-diversity indices of the ileal microbiota revealed that these indices did not change after the early-life GOS intervention. Beta diversity demonstrated overall differences in microbial composition between the CON and GOS groups. The difference between the CON and GOS groups was found on day 8 and day 21, indicating that the ileal microbial composition was modulated by the early-life GOS intervention. This is consistent with the study of Monteagudo-Mera et al. [15]. Current research showed that the impact of early life GOS intervention on the composition of ileal microbiota in piglets of different individuals, that was consistent with the selective response of other intestinal microbiota to prebiotics [15,33]. Additionally, the early-life intervention increased the abundance of Firmicutes and decreased the abundance of Proteobacteria on day 8. These alterations seemed to have a positive influence on the intestinal health of piglets. This may be elucidated by the fact that Firmicutes mainly include SCFA-producing bacteria like *Lactobacillus*, *clostridium,* and *Ruminococcus*, while Proteobacteria mainly include potentially pathogenic bacteria, such as *Escherichia*, *salmonella,* and *Helicobacter pylori*. In addition, we also found that the ratio of Firmicutes to Bacteroidetes increased in the GOS group on day 8 and day 21. Previous study has shown that the Firmicutes/Bacteroidetes ratio increased with an increased body mass index (BMI), which is consistent with our previous results on the improvement of growth performance [31,34]. 

Further analysis at the genus level indicated that the early-life GOS intervention significantly increased the relative abundance of *Lactobacillus* and unclassified Lactobacillaceae on day 8 and day 21, while significantly decreasing the relative abundance of *Streptococcus* and *Clostridium sensu stricto* on day 8 and reducing the relative abundance of *Escherichia*, unclassified Bacteroidales, *Clostridium sensu stricto,* and *Alloprevotella* on day 21. Many studies have reported the impact of these bacteria genera on intestinal health. The genus *Lactobacillus* is the largest genus in lactic acid bacteria, and also the most predominant genus in the small intestine [4,35]. Certain members of the genus *Lactobacillus* affect intestinal physiology, regulate the immune system, and maintain intestinal homeostasis in the host [36]. The benefits of probiotic *Lactobacillus* supplementation for pigs include overall health promotion and growth performance improvement, ultimately increasing the productivity in the swine industry [36,37,38]. In addition, *Lactobacillus* can ferment carbohydrates into lactate and balance the intestinal ecology of the host [39]. Therefore, the increased abundance of *Lactobacillus* is beneficial to intestinal health. Moreover, *Streptococcus* is known as an opportunistic pathogen that induces morbidity in weaning piglets [4]. *Escherichia* is the most common causes of intestinal tract infections in their hosts [40]. In the present study, the reduction of *Streptococcus* and *Escherichia* suggests that the morbidity of piglets may decrease as these bacteria are challenged with weaning. Downes et al. demonstrated that *Alloprevotella* mainly produced succinate and acetate, which could maintain intestinal integrity and inhibit inflammatory response [41]. Moreover, after interferon tau (IFNT) supplementation, the decreased expression of inflammatory cytokines in mouse intestine results in an increased proportion of *Alloprevotella* in the colon, implying that *Alloprevotella* is a kind of beneficial bacteria [42]. However, our results revealed that the abundance of *Alloprevotella* decreased after an early-life GOS intervention, which was mainly due to the increased abundance of *Lactobacillus* competing for the nutrient substrates, leading to a decrease in the proportion of *Alloprevotella*. Additionally, previous studies suggested that *Clostridium sensu stricto* was sharply reduced when dietary crude protein levels were reduced due to the shortage of protein substrates required for fermentation [43,44]. Therefore, the increased abundance of *Lactobacillus* and the decreased abundance of *Clostridium sensu stricto* indicated that the early-life GOS might affect the colonization of intestinal microbiota, enhance the ability to ferment carbohydrates, and reduce the ability to ferment proteins. Overall, the changes caused by the early-life GOS intervention resulted in an improvement in intestinal health.

Some undigested carbohydrates, including cellulose, resistant starch, and oligosaccharides, can be fermented by intestinal microbiota to produce lactate and SCFAs [45]. Previous studies have demonstrated that GOS can be fermented by *Lactobacillus* and *Bifidobacteria* in the intestine, mainly producing lactate and SCFAs [15]. Lactate is a crucial bacterial fermentation product in the small intestine that can reduce the pH value in the small intestine, and inhibit the activity of pathogens [4,46]. In the present study, the lactate concentration increased in the GOS group on day 8, probably due to the increase in lactate-producing bacteria, such as *Lactobacillus*. In addition, consistent with the change of lactate concentration, the pH value in the ileum decreased on day 8. Moreover, SCFAs like acetate, propionate, and butyrate, are main final products from carbohydrates fermentation, which plays an important role not only in regulating intestinal physiology, intestinal development, and nutrient absorption, but also in providing energy to the epithelial cells [47]. In addition, SCFAs can regulate and promote host metabolism when they are absorbed by the intestinal epithelium into the host circulatory system [48]. Acetate and butyrate can be used for lipid biosynthesis, while propionate is mainly involved in the process of hepatic gluconeogenesis [49]. In this study, acetate displayed an increasing trend, and both propionate and butyrate increased significantly, which was beneficial to intestinal growth and health. Additionally, previous studies have shown that *Escherichia* prefers to live in a weakly alkaline environment [43]. Thus, a higher concentration of SCFAs generated from prebiotics GOS supplementation may partly inhibit the proliferation of *Escherichia*. The composition of intestinal microbiota can affect SCFA concentration [49]. Correlation analysis revealed the connection between intestinal microbiota and microbial metabolites in the ileum, suggesting that the increase of *Lactobacillus* and unclassified Lactobacillaceae may result in the enhancement in ileal propionate and butyrate concentrations, and that the decrease of *Clostridium sensu stricto* may promote the increase in ileal propionate and butyrate concentrations. Collectively, the early-life GOS intervention increased the concentration of microbial metabolites, suggesting the increase of microbial carbohydrate fermentation in the GOS group.

The endocrine peptides produced by L-cells can act as intestinal growth factors and play a key role in the development of the intestine. For example, several endocrine peptides can increase the proliferation, differentiation, and apoptosis of intestinal epithelial cells through regulating the secretion of the digestive glands and being involved in the processes of glycolysis and protein synthesis [50,51,52]. In this study, the contents of four endocrine peptides (GLP-1, GLP-2, EGF, and IGF-1) were detected. A previous study demonstrated that nondigestible carbohydrates can stimulate the secretion of intestinal endocrine peptides after being fermented by intestinal microbiota [53]. Consistent with the previous study, a significant increase of GLP-1 concentration and a trend of increase in EGF concentration were observed in the ileum following the early-life GOS intervention in the current study. The correlation analysis results revealed that the abundance of *Lactobacillus* and unclassified Lactobacillaceae was positively correlated with the concentration of endocrine peptides, indicating that GOS stimulate the secretion of GLP-1 and EGF after fermentation by *Lactobacillus* and unclassified Lactobacillaceae. In addition, accumulated evidence suggests that SCFAs activation of GPR41 and GPR43 increases the level of various endocrine peptides [54,55]. Therefore, SCFAs produced by intestinal microbial fermentation of GOS may have been responsible for activating GPCRs in this study, and thereby promoting the secretion of endocrine peptides. The correlation analysis confirmed the relationship between microbial metabolites and endocrine peptides in the ileum.

Intestinal microbiota and their metabolites influence the intestinal immune status. Antimicrobial peptides are expressed in the fetal intestine during pregnancy, and play an important role in innate immunity during early life [56]. Previous studies have shown that being fed a GOS diet for three days increased the mRNA expression of *β-defensin-2* in the colon, which has a protective effect, contributing to the suppression of microbial infections or bacterial outgrowth [57]. Consistent with these previous studies, our results demonstrated that the early-life GOS intervention increased the mRNA expression of antimicrobial peptides, such as *PBD-1* and *PBD-3*. As described in other studies, the increase in antimicrobial peptides indicated a stronger ability to kill specific pathogens by destroying bacterial cell membrane [58]. The correlation analysis showed that the mRNA expression of *PBD-1* and *PBD-3* was negatively related to the abundance of *Fusobacterium* and *Haemophilus*. *Fusobacterium* is associated with lameness and facial skin necrosis in pigs [59]. Many members in genus *Haemophilus* are important human pathogens that cause serious diseases in children and adults [60]. In the present study, the abundance of *Haemophilus* decreased in the GOS group on day 8; however, the difference was not significant, suggesting that piglets were less likely to be invaded by pathogens when weaning in the future. Studies have shown that proinflammatory cytokines play a central role in intestinal inflammatory diseases, and their expression is affected by intestinal symbiotic bacteria [61]. Anti-inflammatory cytokines inhibit the over-activation of immune response and the production of proinflammatory cytokines that preserve the immune homeostasis [62]. IL-8 and IL-10 belong to the family of proinflammatory cytokine and anti-inflammatory cytokine, respectively. In the present study, the early-life GOS intervention did not affect the mRNA expression of *IL-8*, but significantly increased the mRNA expression of *IL-10* on day 21, suggesting a decreased susceptibility to infection by pathogens. Additionally, the correlation analysis revealed that the increase of microbial metabolites may increase the mRNA expression of *IL-10* in ileum. Overall, the changes in the mRNA expression of inflammatory cytokines and antimicrobial peptides caused by GOS treatment are beneficial to intestinal health.

## 5. Conclusions

In this study, we analyzed the effects of early-life GOS intervention on the colonization of ileal microbiota, microbial metabolites, the secretion of endocrine peptides, and the mRNA expression of inflammatory cytokines and the antimicrobial peptides level. In suckling piglets, early-life GOS intervention had beneficial effects on ileal microbiota composition, which was reflected in greater proportions of beneficial and fiber-degrading bacteria (*Lactobacillus*, unclassified Lactobacillaceae) and significantly reduced proportions of potentially pathogenic bacteria (*Clostridium sensu stricto* and *Escherichia*). Furthermore, the early-life GOS intervention markedly increased SCFA and lactate concentrations in the ileum. In addition, the early-life GOS intervention increased concentrations of endocrine peptides and mRNA expression of anti-inflammatory cytokines, which are associated with alterations in ileal microbiota induced by the early-life GOS intervention and their interaction with SCFAs. These findings will facilitate the improvement of approaches for the regulation of intestinal microbiota through early-life GOS intervention to improve newborn health.

## Figures and Tables

**Table 1:** Ileal pH value and concentrations of short-chain fatty acids (SCFAs) and lactate in ileal digesta of piglets on the day 8 and day 21 ^a^.

Item	CON	GOS	*P*-Value
Day 8			
pH value	7.05 ± 0.07	6.57 ± 0.11	0.009
Acetate (μmol/g digesta)	3.73 ± 0.49	10.09 ± 3.21	0.099
Propionate (μmol/g digesta)	0.83 ± 0.57	2.59 ± 0.29	0.000
Butyrate (μmol/g digesta)	0.59 ± 0.10	1.84 ± 0.36	0.011
Valerate (μmol/g digesta)	0.16 ± 0.02	0.25 ± 0.02	0.008
Lactate (μmol/g digesta)	15.15 ± 0.55	18.54 ± 0.66	0.003
Day 21			
pH value	6.94 ± 0.07	7.05 ± 0.11	0.406
Acetate (μmol/g digesta)	9.43 ± 1.88	9.96 ± 1.85	0.847
Propionate (μmol/g digesta)	2.18 ± 0.47	2.18 ± 0.35	0.996
Butyrate (μmol/g digesta)	0.73 ± 0.08	1.13 ± 0.14	0.041
Valerate (μumol/g digesta)	0.29 ± 0.05	0.30 ± 0.06	0.939
Lactate (μmol/g digesta)	29.53 ± 1.40	30.28 ± 1.20	0.693

^a^ Values are means ± SEM, *n* = 6; CON, a control group; GOS, a galacto-oligosaccharides intervention group; SCFAs, short-chain fatty acids.

**Table 2:** The concentrations of endocrine peptides in ileal mucosa of piglets on the day 8 and day 21 ^a^.

Item ^b^	CON	GOS	*P*-Value
Day 8			
GLP-1 (pg/g)	1.16 ± 0.10	1.51 ± 0.10	0.033
GLP-2 (pg/g)	2.25 ± 0.23	2.66 ± 0.14	0.155
IGF-1 (ug/g)	4.38 ± 0.57	5.37 ± 0.30	0.156
EGF (ng/g)	114.63 ± 10.05	153.99 ± 15.50	0.059
Day 21			
GLP-1 (pg/g)	1.47 ± 0.13	1.69 ± 0.08	0.163
GLP-2 (pg/g)	2.83 ± 0.17	3.06 ± 0.26	0.463
IGF-1 (ug/g)	5.24 ± 0.33	6.15 ± 0.60	0.216
EGF (ng/g)	154.19 ± 8.90	167.18 ± 15.84	0.491

^a^ Values are means ± SEM, *n* = 6; CON, a control group; GOS, a galacto-oligosaccharides intervention group. ^b^ GLP-1, glucagon-like peptide-1; GLP-2, glucagon-like peptide-2; IGF-1, insulin-like growth factor 1; EGF, epidermal growth factor.

## Supplementary Materials

The following are available online at https://www.mdpi.com/2072-6643/11/8/1753/s1, Figure S1: The composition of GOS, Table S1: The information of carbohydrate components in GOS sample, Table S2: Primer sequences for quantitative real-time PCR analysis, Table S3: The average clean data acquired during sequencing, Table S4: Relative abundance of the main phylum, Table S5: Relative abundance in the top 20 genera.
