# Supplementary material for: Changes in Ileal Microbial Composition and Microbial Metabolism by an Early-Life Galacto-Oligosaccharides Intervention in a Neonatal Porcine Model
Source: Nutrients. 2019 Jul 30;11(8):1753. doi: 10.3390/nu11081753 (PMC6723927; doi:10.3390/nu11081753)
Supplement: Supplementary file 1 [file nutrients-11-01753-s001.pdf]

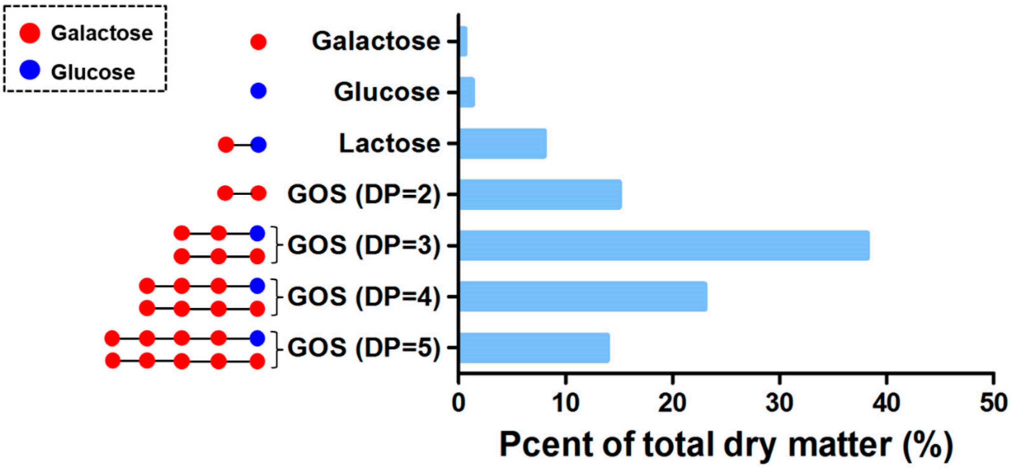

Figure S1. The composition of GOS. The composition of GOS was measured using High Performance Ionic-Exchange Chromatography (HPIEC). The abundance of each oligosaccharide is presented as percent of the total amount of oligosaccharides.

Table S1. The information of carbohydrate components in GOS sample.

Items	Molecular formula	Molecular weight
Galactose	$C_6H_{12}O_6$	180.15
Glucose	$C_6H_{12}O_6$	180.15
Lactose	$C_{12}H_{22}O_{11}$	342.28
GOS (DP=2)	$C_{12}H_{22}O_{11}$	342.28
GOS (DP=3)	$C_{18}H_{32}O_{16}$	540.42
GOS (DP=4)	$C_{24}H_{42}O_{21}$	666.56
GOS (DP=5)	$C_{30}H_{52}O_{26}$	828.70

Table S2. Primer sequences for quantitative real-time PCR analysis.

Gene[a]	Nucleotide sequences 5’-3’	Product length/bp	Accession number
IL-8	ACTGGCTGT TGCCTTCTT/CAGTT CTCTTCAAAAATATCTG	278	NM_213867.1
IL-10	GTCCGACTCAACGAAGAAGG/GCCAGGAAGATCAGGCAATA	73	NM_214041.1
PBD-1	GGCAAGTGTGCTCCAAAGATG/ CTGAGCCATATCTGTGGGGTT	113	NM_213838.1
PBD-3	TGCCTTGCTCTTCTTGTTCCT/ TTTTCGGCCACTCACAGAAC	160	NM_214444.1
Reg-3γ	GATTCCCCAGCAGACACGC/ GACACGAAGGATGCCTCAGC	185	NM 001144847.1
β-actin	ATGCTTCTAGACGGACTGCG/GTTTCAGGAGGCTGGCATGA	109	XM_003357928.4

[a] *IL-8*, interleukin-8; *IL-10*, interleukin-10; *PBD-1*, porcine β-defensin-1; *PBD-3*, porcine β-defensin-3; *Reg-3γ*, regenerating islet derived protein-3γ.

Table S3. The average clean data acquired during sequencing

Item	groups	raw data	valid data	valid%	Q20%	Q30%	GC%
Day 8	CON	34683	33537	96.70	95.61	86.41	51.97
	GOS	27782	26734	96.06	95.05	85.20	52.28
Day 21	CON	35030	33653	96.09	95.15	85.45	52.35
	GOS	32069	31133	97.16	95.64	86.45	53.42

The number of average raw sequences detected in a group was at least 27782 reads, with 26734 valid sequences.

The indexes of quality control Q20 and Q30 were sufficient to ensure the accuracy of sequencing.

CON, a control group; GOS, a galacto-oligosaccharides intervention group.

Table S4 Relative abundance of the main phylum.

Items	CON	CON	CON	CON	CON	GOS	GOS	GOS	GOS	GOS
Day 8										
Firmicutes	30.61015	36.96849	72.82878	60.17529	67.67553	98.02896	95.03074	98.09584	96.66012	96.8037
Proteobacteria	66.63242	59.35616	23.14556	1.655976	14.13088	1.158207	1.804358	0.833399	1.178782	1.223209
Bacteroidetes	0.155768	0.087974	0.089752	33.27654	0.623722	0.139543	0.243734	0.160878	0.649841	1.239164
Fusobacteria	2.270242	2.691343	0.678422	0.011136	17.38923	0.397698	1.291426	0.026373	0.770742	0.122321
Actinobacteria	0.288337	0.325828	2.035267	0.717181	0.143149	0.202337	1.462403	0.86241	0.297214	0.356326
Candidatus Saccharibacteria	0.036456	0.524584	1.169421	0.053454	0.003408	0.066283	0.105497	0.002637	0.377815	0.090411
Others	0.006628	0.045616	0.052796	4.110428	0.034083	0.006977	0.061843	0.018461	0.065488	0.164867
Day 21										
Firmicutes	67.48153	71.14274	90.54933	78.72503	48.14966	92.48467	97.30887	86.646	81.49717	70.7187
Proteobacteria	29.72576	24.59741	0.249305	7.968543	2.428099	0.808253	0.082749	5.816668	2.375203	22.85042
Actinobacteria	0.150853	2.668861	8.384675	3.437957	20.76992	5.937821	2.525634	0.612671	15.24484	5.552989
Bacteroidetes	2.115809	0.755155	0.74505	6.374835	22.9015	0.350952	0.007196	5.213879	0.407743	0.050405
Fusobacteria	0.421615	0.751928	0.063043	1.792052	0.003818	0.219788	0.017989	0.1087	0.063339	0.09661
Candidatus Saccharibacteria	0.007736	0.048407	0.005731	0.222702	0.278697	0.070899	0.03238	0.128463	0.324611	0.609065
Others	0.096701	0.035499	0.002866	1.478878	5.468313	0.127619	0.025184	1.473622	0.087091	0.121813

CON, a control group; GOS, a galacto-oligosaccharides intervention group.

Table S5 Relative abundance in the top 20 genera.

Items	CON	CON	CON	CON	CON	GOS	GOS	GOS	GOS	GOS
Day 8										
Lactobacillus	6.1279952	8.885341	32.841455	58.030425	53.847989	95.548578	84.812107	95.018066	89.859453	92.229963
unclassified Porphyromonadaceae	0.0033142	0.0032583	0.0052796	21.802753	0.0068166	0	0.0181891	0.1081309	0.0302252	0.0744562
Fusobacterium	2.2602989	2.6326936	0.4777995	0.0033409	17.372188	0.1918716	1.0949834	0.0210987	0.5289406	0.106366
Actinobacillus	49.938687	47.486234	1.863682	0.0055682	9.0797546	0.3069946	0.582051	0.2399979	0.0906755	0.0159549
Romboutsia	9.3825606	21.436252	14.843461	0.0111364	2.5732788	0.0174429	3.4668413	0.4615344	0.0604504	0.0904111
Veillonella	8.8025718	0.8276042	0.506837	0.0100227	3.4730743	0.1814059	1.4587653	0.698895	0.7858546	0.1701856
Barnesiella	0	0.0032583	0	5.3020179	0.0238582	0	0	0.015824	0.0151126	0.0744562
unclassified Ruminococcaceae	0.0231995	0.0423577	0.1082308	0.1392044	1.4110429	0.0523286	0.1418749	0.0474721	2.4230517	1.3189385
unclassified Bacteria	0.0033142	0	0.0131989	4.0781327	0	0	0	0.0026373	0	0
unclassified Pasteurellaceae	9.299705	1.5998175	0.2059025	0.0033409	2.2597137	0.0418629	0.1309615	0.0316481	0.0554128	0.0053183
Streptococcus	0.9114109	1.6291421	10.704292	1.1861155	2.4191495	0.3385451	1.0633947	0.6408735	0.7254043	0.5265117
Escherichia	0.7092434	0.0423577	20.054379	0.01225	0.872529	0.0104657	0.1527884	0.1002189	0.1763135	0.9200659
unclassified Bacteroidales	0	0	0.0026398	2.1392935	0.0068166	0	0	0.0052747	0	0.0106366
Terrisporobacter	0.3711928	1.0068098	1.0083945	0.0222727	1.2269939	0.0104657	0.2764742	0.3507661	0.1158632	0.053183
Haemophilus	6.4859311	8.7778176	0.4698802	0.0011136	1.4689843	0.02442	0.0218269	0.0501094	0	0
unclassified Lachnospiraceae	0.2319955	0.091232	0.9292012	0.5746358	0.2010907	0.04884	0.1309615	0.0553841	0.2216513	0.2286869
unclassified Clostridiaceae 1	2.2934412	0.6060409	0.5860303	0.0267272	0.7736878	0	0.0327404	0.0553841	0.0403002	0.0850928
unclassified Lactobacillaceae	0.0198853	0.0162914	0.1319888	0.1581362	0.2283572	0.2825746	0.3710575	0.2927447	0.3475895	0.4733287
Clostridium sensu stricto	0.7390714	0.2020136	0.1715854	0.0189318	1.1042945	0.0139543	0.0509295	0.0052747	0	0.0265915
Alloprevotella	0.0132569	0.0162914	0	1.5167713	0	0.0069771	0.0145513	0.0026373	0.0352627	0
Others	2.3829251	4.6951875	15.075762	4.9578108	1.6503802	2.9232724	6.1795025	1.7960282	4.4884389	3.5898527
Day 21										
Lactobacillus	52.191235	29.412334	40.109465	52.446238	21.449478	85.167854	93.103076	60.816236	61.117929	41.958248

unclassified Porphyromonadaceae	0.3751982	0.0096815	0.0085967	0.8908066	16.667091	0.2304229	0.0071955	1.6354361	0.0197934	0
Fusobacterium	0.3171779	0.7454739	0.0487148	1.1239474	0.0025452	0.2126981	0.0179888	0.0914065	0.0633387	0.0966102
Actinobacillus	19.181526	15.27092	0.0057312	3.6223815	0	0.0425396	0.0035978	0.0765839	0.518586	19.175033
Romboutsia	3.2607434	18.420628	24.712726	1.2005011	0.3245101	1.3648126	0.8922468	0.0247045	1.2153121	11.072374
Veillonella	1.9108034	3.6273276	1.1061123	0.3131742	0.0292695	0.3190471	0.0179888	0.1334042	6.1082301	0.6426681
Barnesiella	0.3597261	0	0	0.4662816	2.4688216	0.0070899	0	0.2062823	0.0039587	0
unclassified Ruminococcaceae	1.2532395	0.5357085	1.0373385	3.8764006	1.0893357	0.2339679	0	1.3562756	0.0791734	0.0882094
unclassified Bacteria	0.0232081	0.0161358	0	0	5.287605	0	0	0.1815779	0.0158347	0.113412
unclassified Pasteurellaceae	2.9358295	6.3994578	0.0028656	0.1879045	0	0.0106349	0.0035978	0.0247045	0.0435454	1.0207082
Streptococcus	0.1469849	8.1647142	1.8425653	0.5741527	1.518198	1.0953951	0.9030401	3.5339748	6.4882625	7.7498215
Escherichia	0.5956756	0.9165134	0.114623	0.4454033	0.1030797	0.4324861	0.0035978	0.0098818	0.0712561	0.0882094
unclassified Bacteroidales	0.1237767	0.3582147	0.1232198	0.497599	1.3133113	0	0	0.24828	0.0039587	0
Terrisporobacter	4.1349166	1.2844096	11.44511	1.4406013	1.0473403	1.4817966	0.6835762	0.2013415	0.1979336	1.3525434
Haemophilus	4.4985108	1.3747701	0.0028656	0.0765537	0	0	0	0.0074113	0.0158347	0.0504053
unclassified Lachnospiraceae	0.0812285	1.1746862	0.1346821	6.249565	12.090863	0.0567195	0.0215866	13.736922	0.1979336	0.4494476
unclassified Clostridiaceae 1	1.2957877	1.6813502	3.9831504	0.0452363	1.1084245	0.1985182	0.0503688	0.071643	0.2058509	0.9787037
unclassified Lactobacillaceae	0.170193	0.1355407	0.0429836	0.1983437	0.086536	0.4183062	0.4749056	0.2186346	0.2454376	0.1386147
Clostridium sensu stricto	1.0559703	0.4130765	0.5559217	0.2087828	0.7279206	0.3438619	0.3993524	0.0382919	0.1227188	0.0798085
Alloprevotella	0.3017058	0.0129086	0.0573115	1.6772218	0.7864597	0	0	0.0839952	0.0079173	0
Others	5.7865625	10.046148	14.666017	24.458905	33.899211	8.3838491	3.4178809	17.303013	23.257195	14.945184

CON, a control group; GOS, a galacto-oligosaccharides intervention group.
